# Supplementary material for: Identification of photoperiod-induced specific miRNAs in the adrenal glands of Sunite sheep (Ovis aries)
Source: Front Vet Sci. 2022 Jul 22;9:888207. doi: 10.3389/fvets.2022.888207 (PMC9354845; doi:10.3389/fvets.2022.888207)
Supplement: Supplementary file 1 [file Data_Sheet_1.ZIP › Supplementary file/miRDeep2 file.zip!/miRDeep2 file/Novel_Structure/SP42A2/Novel_451.pdf]

Provisional ID	:	NC_019479.2_37808
Score total	:	1.9
Score for star read(s)	:	-1.3
Score for read counts	:	0
Score for mfe	:	1.6
Score for randfold	:	1.6
Score for cons. seed	:	
Total read count	:	61
Mature read count	:	61
Loop read count	:	0
Star read count	:	0

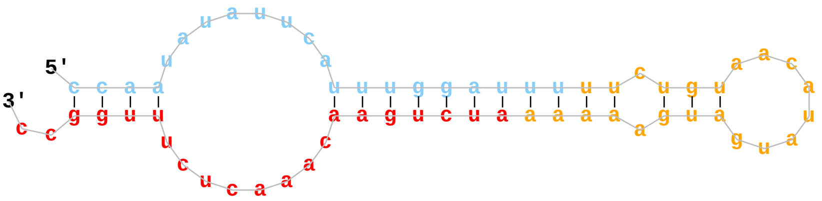

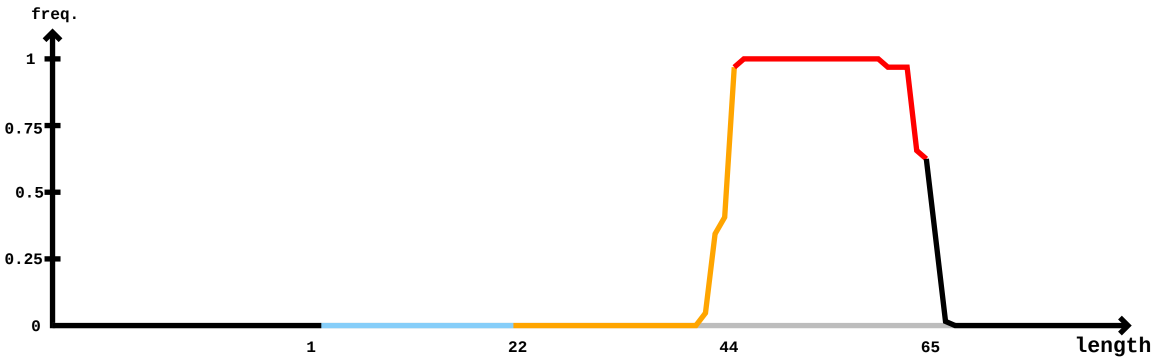

Star Mature

```
5'- agaucuggaacaagguauuggguuggccaauauauucauuuggauuuuucuguaacauaugaugaaaaaaucugaacaaacucuuuggccaacccaauacuaugaggaug   -3'    exp
    ..((((....((.(((((((((((((((((........(((((((((((.(((........))).)))))))))))........))))))))))))))))).)).)))).    reads      mm         sample
    ..................................................................aaaaucugaacaaacuUuu.........................    2          1          seq
    ..................................................................aaaaucugaacaaacucuuugg......................    1          0          seq
    ...................................................................aaaucugaacaaacuUuuugg......................    19         1          seq
    ....................................................................aaucugaacaaacuUuuuggc.....................    2          1          seq
    ....................................................................aaucugaacaaacuUuuuggcc....................    2          1          seq
    .....................................................................aucugaacaaacuUuuuggcc....................    25         1          seq
    .....................................................................aucugaacaaacuUuuuggcca...................    11         1          seq
    ......................................................................ucugaacaaacucuuuggcc....................    1          0          seq
    ......................................................................ucugaacaaacuUuuuggccaa..................    1          1          seq
```
